# Physiological and Psychological Resilience Among Healthcare Workers in COVID–19 Units—The Protective Role of Religious Beliefs

**DOI:** 10.1002/ijop.70047

**Published:** 2025-04-06

**Authors:** Einat Mader, Janne L. Punski‐Hoogervorst, Hernan Kosovsky, Aaron Pinkhasov, Morgan Peltier, Boaz Bloch, Avi Avital

**Affiliations:** ^1^ Medicine and Psychiatry Service Emek Medical Center Afula Israel; ^2^ Department of Occupational Therapy Faculty of Social Welfare and Health Sciences, University of Haifa Haifa Israel; ^3^ Department of Psychiatry Emek Medical Center Afula Israel; ^4^ Department of Medicine and Psychiatry NYU Langone Hospital – Long Island, NYU Long Island School of Medicine New York USA; ^5^ Department of Psychiatry and Behavioral Health Jersey Shore University Medical Center Neptune New Jersey USA

**Keywords:** attentional dysregulation, auditory sustained attention test, emotional dysregulation, religiosity, resilience

## Abstract

The COVID‐19 pandemic profoundly impacted global health, with disproportionate consequences for healthcare workers (HCWs). Religious beliefs and practices may improve psychological resilience by fostering community, providing purpose and giving meaning to hardships. Yet, how religiosity impacts HCWs during a time of crisis is unclear. We therefore performed a cross‐sectional study to investigate how religiosity contributes to resilience among HCWs who were routinely exposed to high levels of stress during the pandemic, through a physiological measure (the Auditory Sustained Attention Test; ASAT) and psychological self‐reports. Forty‐two HCWs were recruited from COVID‐19 units and 44 HCWs from general internal medicine units during June and July 2022. COVID‐19 HCWs showed significantly elevated emotional and attentional dysregulation with the ASAT, as measured by acoustic startle and prepulse inhibition, that was undetectable with self‐reports. Furthermore, after dividing the HCWs into a ‘high’ and ‘low’ religiosity group, those in the ‘low’ group showed higher emotional and attentional dysregulation with the ASAT. Findings suggest that the ASAT has greater sensitivity at detecting emotional and attentional dysregulations than self‐reports. Moderate or high religiosity may lead to better performance on the ASAT which could suggest greater resilience to mental health problems in the face of a crisis.

## 1 Introduction

On December 31, 2019, the World Health Organisation (WHO) was informed about increasing cases of pneumonia with unknown aetiology in the city of Wuhan, China. Within a week, it became clear that these cases were caused by a novel coronavirus that was soon named severe acute respiratory syndrome coronavirus 2 (SARS‐CoV‐2). The WHO declared the outbreak a pandemic on March 11, 2020 (World Health Organization 2020).

Although the COVID‐19 pandemic affected many people globally, an enormous strain was placed on healthcare workers (HCWs). HCWs were at high risk of contracting the disease, as the healthcare system was largely unprepared with widespread shortages of personal protective equipment and inadequate protective measures (Smallwood et al. 2022). Furthermore, there were large numbers of cases and few, if any, evidence‐based guidelines regarding clinical management of patients (Filip et al. 2022). Increasing numbers of school and work closures were employed as governments struggled to limit the spread of the virus that created additional problems at home, such as managing remote work while supervising children and coping with household stressors (Lachance et al. 2022).

Throughout these challenges, HCWs remained committed to providing patient care, often putting their own physical and mental health at risk. HCWs experienced an elevated prevalence of mental health problems (MHP) such as depression, anxiety, posttraumatic stress disorder (PTSD), moral injury and insomnia among those who worked on the frontlines during the COVID‐19 pandemic (Saragih et al. 2021; Vindegaard and Benros 2020). The increased prevalence of these mental health challenges placed an additional burden on HCWs that already had high rates of anxiety, depression and suicide (Mata et al. 2015). A meta‐analysis of 70 cross‐sectional studies from the US, Asia and Europe showed a pooled prevalence of acute stress at 56% (95% CI: 30–82) and sleep disturbances in 44% (95% CI: 25–64) (Marvaldi et al. 2021). Another meta‐analysis assessing MHP among HCWs during the COVID‐19 pandemic found the prevalence rates to be as high as 49% (95% CI: 22–75) for PTSD, 40% (95% CI: [CI]: 29–52) for anxiety symptoms, 37% (95% CI: 29–45) for depression and 37% (95% CI: 25–50) for general distress (Saragih et al. 2021). These numbers are higher than a previous meta‐analysis (Salari et al. 2020), which could be attributed to reasons such as differences in study populations, outcome measures and methodological approaches. While Marvaldi et al. included a range of cross‐sectional studies from diverse geographical regions and focused on acute stress and sleep disturbances, Saragih et al. specifically assessed mental health outcomes among HCWs during the COVID‐19 pandemic, likely capturing the heightened psychological burden in this group.

Beyond prevalence data, it is crucial to understand the factors influencing mental health outcomes among HCWs. Among the risk factors that have been associated with MHP among HCWs during the COVID‐19 pandemic are: female gender, current or past medical condition, worrying about the risk of infection to family, friends and acquaintances, working at the frontline with direct care for COVID‐19 patients, lack of trust in one's own ability to cope, changes to regular job duties, and working overtime in high‐stress environments (Saragih et al. 2021; Vindegaard and Benros 2020). While it is crucial to identify risk factors associated with MHP among HCWs at the frontlines of the COVID‐19 pandemic, it is equally important to investigate protective or resilience factors that may explain why some HCWs develop symptoms such as anxiety and depression while others do not.

### 1.1 Resilience

Psychological resilience is the ability to remain in an emotional and cognitive equilibrium with relatively stable levels of functioning, despite exposure to adverse events that for others may give way to anxiety, depression or other mental health disorders (Fletcher and Sarkar 2013; Windle 2011). While the term ‘recovery’ suggests the ability to overcome temporary (sub)threshold symptoms of psychopathology, resilience reflects stable psychological and physical function through mental, behavioural, and emotional flexibility that allows a successful adaptation to challenging life experiences (Windle 2011). It is a dynamic process that results from a complex interplay of various protective factors, such as hardiness, positive emotions, self‐efficacy and spirituality. These factors are frequently divided into personal resilience that consists of individual traits and community resilience that reflects support networks and shared resources (Fletcher and Sarkar 2013; Rutter 2012). Resilience is therefore considered both a mental process and a personality trait (Windle 2011).

Identification of resilience factors is difficult because their effectiveness can vary depending on time and circumstances; certain factors may enhance resilience in certain situations but not in others, depending on the type, intensity and duration of stressors. Additionally, resilience factors may interact differently across varying life stages, cultural contexts, or environments, making their identification and generalisation complex. (Rutter 2012; Windle 2011).

In the context of the COVID‐19 pandemic, studies aiming to ascertain mechanisms of resilience among HCWs found a few factors that may improve psychological resilience. These included: quality of sleep, emotion‐centred coping, good self‐esteem and self‐compassion, adequate social and organisational support, life satisfaction, work experience and involvement in policy development (Labrague 2021). Most of these studies used subjective self‐administered patient questionnaires. Therefore, the data may be either exaggerated or underreported due to social desirability bias or a lack of introspection. Previous research from our lab has shown that individuals who are able to better sustain attention during a neurophysiological auditory task—an indicator of focus and reduced distractibility—may be more resilient to stress and adversity (Cohen et al. 2024; Dolev et al. 2021), suggesting a method to objectively measure components of resilience.

Another important yet less explored aspect of resilience is the role of religious beliefs, which are known to have a positive impact on well‐being—especially in situations of stress or adversity (Park 2005; Pargament 1997), and religious practice may enhance resilience (Ano and Vasconcelles 2005; de Diego‐Cordero et al. 2022). The influence of religious beliefs on the resilience of HCW in the context of the COVID‐19 pandemic, however, has not been widely studied. A study investigating the influence of Christian/Catholic and Buddhist/Taoist beliefs on resilience in Taiwanese HCWs found that religious beliefs increased the level of happiness and reduced the level of mental distress (Edara et al. 2021). Similarly, research among Brazilian HCWs revealed a significant association between faith and reduced acute anxiety during the COVID‐19 outbreak (Tolentino et al. 2022). Finally, it was shown that spiritual/religious practices reduced mental distress and increased psychological resilience among Iranian and Turkish HCWs (Angin 2021; Mesri et al. 2022). These studies, like others, were based on subjective self‐assessment tools and did not account for diverse religious beliefs within and across different religions in their respective populations. Accounting for such diversity is important because religious frameworks can significantly influence how individuals perceive, interpret, and cope with adversity (Pargament 1997). For instance, beliefs about suffering, forgiveness, or divine support may shape mental health outcomes and responses to stress differently across religious groups (Ano and Vasconcelles 2005; Pargament 1997). Failing to consider this diversity limits the generalisability of findings and overlooks the potential role of coping mechanisms related to religious beliefs.

### 1.2 Research Aims

We aimed to measure resilience (personal and community), mental health difficulties (anxiety and sleep disturbances) and religiosity (religious beliefs and religious practices) among Israeli HCWs, using both self‐assessment reports and neurophysiological tools. The primary objective was to demonstrate the differences in self‐reported resilience between HCWs who report high levels of religiosity versus those who report low levels of religiosity. The additional objective was to compare the self‐reported resilience factors among HCWs in COVID‐19 departments to those working in general internal medicine departments. We hypothesised that HCWs who report high levels of religiosity would be more resilient than HCWs who report low levels of religiosity. Finally, we hypothesised that deploying objective physiological measures of resilience such as the ASAT would allow us to detect emotional and attentional dysregulation and differentiate those between COVID‐19 and non‐COVID‐19 HCWs depending on their level of religiosity.

## 2 Subjects and Methods

### 2.1 Overview

**FIGURE 1:**
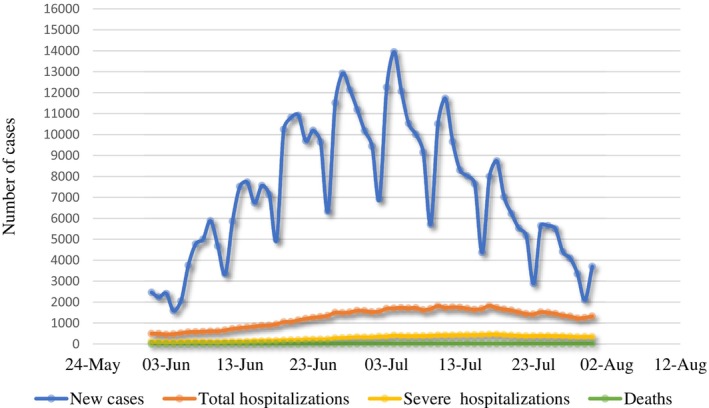
COVID‐19 status in Israel between June 1 and July 31, 2022.

This cross‐sectional study was conducted at the Emek Medical Center (MC): a regional, academic hospital that serves approximately 700,000 residents in northeastern Israel. Data was collected between June and July 2022. In the period between the first confirmed case of COVID‐19 on February 21, 2020, and the start of the research, Israel had seen three domestic lockdowns with a total duration of approximately 4 months and a significant ban on international travel for over 2 years. Although the pandemic had passed its initial stages and the most severe peak of daily cases (between January and April of 2022, with a maximum daily cases of 83,772 on a population of just over 9 million) and vaccination rates were relatively high (namely as of June 1, 2022: 75% of the population with a first dose, 66% with a second, 48% with a third and 9% with a fourth) (Israel Ministry of Health 2023), the state of emergency and uncertainty was ongoing. Driven by new variants of Ba‐4 and Ba‐5, daily cases, hospitalisations and deaths were yet again climbing (see Figure 1).

### 2.2 Subjects

The study population consisted of Emek MC employees. Participants had to be 18 years or older and work in COVID‐19 units or another area of the hospital during the inclusion period and the 6 months before. Exclusion criteria were any uncorrected hearing disorder due to the auditory nature of the ASAT.

### 2.3 Ethical Considerations

This project was performed according to the Declaration of Helsinki guidelines with prior approval and oversight from the Emek MC Institutional Review Board (Protocol# 0017‐22‐EMC). All data were collected during a single session and anonymously (each participant was assigned a numeric code) to increase compliance and maintain discretion. Furthermore, the principal investigator did not recruit participants from her department to minimise the possibility of coercion.

### 2.4 Physiological Tool

Auditory sustained attention refers to the ability to maintain focus on an auditory task or stimulus for an extended period. The Auditory Sustained Attention Test (ASAT) measures physiological attention and anxiety through an automated human monitoring system (Mindtension ltd, Israel) that is used to deliver acoustic startle stimuli and record electromyography (EMG) activity of the orbicularis oculi muscle. Three electrodes with a 4‐mm recording area (EL254, Biopac Systems Inc., CA, USA) were attached to adhesive sticker disks (ADD204, Biopac Systems Inc., CA, USA) and filled with saline electrode gel (SIGNAGEL, Parker Labs, NJ, USA). Two electrodes were then placed approximately 2 cm below the pupil on the orbicularis oculi muscle, and a third (reference) electrode was placed on the mastoid bone. The participants wore headphones (HD400S, Sennheiser, Germany) throughout the trial session to receive the auditory input.

The total duration of the ASAT was 6 min. Each session started with a 1‐min acclimatisation period with a 60 dB (dB) background noise, delivered continuously throughout the session. Next, 26 auditory trials were delivered. These consisted of: eight randomly delivered trials of a single 30 milliseconds (ms) 114 dB ‘pulse alone’ startle stimulus to evaluate individual startle responses (i.e., anxiety level); 2 ‘pre’ stimuli trials of a single 86 dB pulse; 8 ‘pre‐pulse’ trials that consisted of a single 114 dB pulse preceded—after a 120 ms inter‐stimulus‐interval—by a 20 ms pre‐pulse of 26 dB above background noise (i.e., 86 dB); and then 8 trials of ‘no stimulus’ to measure baseline EMG activity and noise levels.

The stimuli delivery and analysis were carried out using the Mindtension software (Mindtension, Israel). Data were recorded at a 1 kHz sampling rate with a Band‐Pass Filter of 10–300 Hz. For each trial, data analysis was performed on the first 300 ms time window that included the EMG maximal peak. The auditory sustained attention was calculated as the percentage of response inhibition:

$$100-\left(\frac{\left(\text{maximum response to}\;\text{‘}\mathrm{pre}‐\text{pulse trial}\text{’}\right)}{\left(\text{maximum response to}\;\text{‘}\text{pulse}\;\text{alone}\text{’}\;\text{trial}\times 100\right)}\right)$$

### 2.5 Self‐Assessment Report Tools

A demographic questionnaire was distributed to collect basic socio‐demographic information. In addition, five self‐report questionnaires were used to gain insight into psychological symptoms (anxiety and sleep disturbances), resilience (personal and community), and level of religiosity. For each questionnaire, the Hebrew‐language version was used.

The Generalised Anxiety Disorder 7‐Items Scale (GAD‐7) is a self‐report screen for generalised anxiety disorder (GAD). It contains seven statements that relate to GAD symptoms and their impact over the last 2 weeks, for example, ‘… how often have you been bothered by… not being able to stop or control worrying?’. Participants answer on a 3‐point Likert scale the extent to which they experience certain symptoms or limitations (0 = not at all to 3 = almost every day). Scores higher than 10 are considered at risk and scores higher than 15 high‐risk for developing a GAD. The GAD‐7 has validity and internal consistency (Cronbach's alpha = 0.92 and intraclass correlation = 0.83) (Spitzer et al. 2006).

The Pittsburgh Sleep Quality Index (PSQI) is a questionnaire designed to quantify the patterns and quality of sleep over a 1‐month time frame. The questions relate to sleep duration, sleep disturbances, sleep onset latency, daytime dysfunction, habitual sleep efficiency, sleep quality and use of sleep medication. An example is: ‘During the past month, how long (in minutes) has it usually takes you to fall asleep each night?’ Answers are given on a 4‐point Likert scale (0 = not during the past month to 3 = three or more times a week). A global sum of 5 or more indicates poor sleep (Crohnbach's alpha = 0.83) (Buysse et al. 1989).

The Connor and Davidson Personal Resilience Scale (CD‐PRS) is a 10‐item measure of personal resilience, based on how the participant has felt over the past month. The CD‐PRS proposes for example ‘I believe I can achieve my goals, even if there are obstacles’. Items are scored on a 5‐point range of responses (0 = not true at all to 4 = true nearly all of the time). Higher scores reflect a greater personal resilience facet (Connor and Davidson 2003). For this research, the 2007 refined version by Campbell‐Sills and Stein was used, that has been tested with a good internal consistency and construct validity (Cornbach's alpha = 0.85) (Campbell‐Sills and Stein 2007).

A COVID‐19 adapted version of the Conjoint Community Resiliency Assessment Measure (CCRAM; Cohen et al. 2024) was used for this study. This questionnaire contains 10 items related to six constructs of community resilience, namely: leadership, collective efficacy, preparedness, place attachment, social trust and social relationship. An example of a question in the CCRAM is ‘My town is organized for emergency situations’. Adaptations were made by adding the statement ‘during the Corona crisis’ to the first question and the statement ‘including the Corona crisis’ to questions 3, 7 and 8. Participants answer on a 5‐point Likert scale the extent to which they agree with each statement (1 = strongly disagree to 5 = strongly agree). The original CCRAM was developed in Israel and has been shown to be a valid tool for measuring community resilience with high internal validity (Crombach's alpha = 0.92) (Leykin et al. 2013).

The Hamada Religiosity Questionnaire (HRQ) is a 3‐item self‐report questionnaire that tests the individual's degree of belief in religious values and the degree to which he or she strives to behave in accordance with these values. The questions are: (i) do you believe in religious values?, (ii) do you behave according to the values of the religious tradition? and (iii) do you observe the religious commandments? Each item is scored on a 5‐point Likert scale (1 = do not agree at all to 5 = agree to a very large extent). The reliability has been tested with a Crombach's alpha = 0.93 (The Shalem Fund 2023).

### 2.6 Procedures

The study was conducted in the Emek MC (Afula, Israel) and approved by the local Review Board (0017‐22‐EMC). Data were collected anonymously and in accordance with the Declaration of Helsinki. First, informed written consent to participate in the study was obtained from all participants. Then, participants were requested to fill in the questionnaires (i.e., the socio‐demographic questionnaire, GAD‐7, PSQI, CD‐PRS, CCRAM and HRQ) and undergo the physiological measure (i.e., ASAT). All tests were conducted between 8:00 am and 12:00 pm to minimise variations in attention and cognitive performance associated with the circadian rhythm; conducting tests during this time ensures more consistent results by controlling for potential declines in attention and cognitive function later in the day. Participants were instructed not to consume nicotine and caffeine throughout the testing period. All data were coded and stored in the computerised systems of Emek MC.

### 2.7 Data Analysis

Descriptive statistics were calculated for all variables to summarise central tendencies and distributions across groups. Religiosity levels were categorised as high or low based on the median HRQ score, with scores ≥ 9 indicating high religiosity. Demographic group differences were compared using Student's *t*‐tests, *χ* ^2^‐tests or Fisher's exact tests—as appropriate—and those between survey scores with generalised linear models. Physiological data were evaluated using linear mixed effects models for the effect of stimuli (decibels), religiosity level, unit and all interactions as fixed effects and HCW differences as random effects. Additional models were also fit to evaluate the impact of potential confounding variables that were identified in univariate analyses as having *p* values < 0.1. Residuals of all models were examined for compliance with the assumptions of parametric techniques (normality, identity and independence of errors) and log‐transformed when appropriate for hypothesis testing. Results are reported as crude and adjusted slopes (differences from control) with 95% confidence intervals or least square means with standard errors. *p* < 0.05 was considered statistically significant. All analyses were performed using R programming.

## 3 Results

### 3.1 Demographics, Religious Association and Professional Occupation

Eighty‐six HCWs were included for participation in the study: forty‐two (49%) worked in COVID‐19 departments and the other forty‐four (51%) in non‐COVID‐19 general internal medicine units. HCWs on the COVID‐unit were more often male (47.6% vs. 36.4%) and, as expected, had more experience working with COVID. HCWs on the COVID‐unit were also more likely to report that religion was important or very important than HCWs on the control unit, though there were no statistically significant differences in religiosity level. No other differences between the populations were detected (see Table 1).

**TABLE 1:** Characteristics of the study participants.

	Control (*n* = 44)	COVID‐unit (*n* = 42)	*p*
Age			0.119
20–30	21 (47.7%)	15 (35.7%)	
30–40	19 (43.2%)	17 (40.5%)	
40–50	2 (4.5%)	9 (21.4%)	
50–60	2 (4.5%)	1 (2.4%)	
Sex			< 0.001***
Female	28 (63.6%)	22 (52.4%)	
Male	16 (36.4%)	20 (47.6%)	
Years working in COVID‐19 department			< 0.001***
0	14 (31.8%)	0 (0%)	
< 0.5	25 (56.8%)	5 (11.9%)	
0.5–1.0	3 (6.8%)	5 (11.9%)	
1–2	2 (4.5%)	10 (23.8%)	
> 2	0 (0%)	21 (50%)	
Unknown	0 (0%)	1 (2.4%)	
Marital status			0.664
Married	21 (47.7%)	26 (61.9%)	
Not married	22 (50.0%)	16 (38.1%)	
Unknown	1 (2.3%)	0 (0%)	
Children			1.000
No	19 (43.2%)	19 (45.2%)	
Yes	24 (54.5%)	23 (54.8%)	
Unknown	1 (2.3%)	0 (0%)	
Profession			0.196
Doctor	14 (31.8%)	13 (31.0%)	
Nurse	13 (29.5%)	20 (47.6%)	
Other	14 (31.8%)	6 (14.3%)	
Unknown	3 (6.8%)	3 (7.1%)	
Religion			0.129
Jewish	21 (47.7%)	10 (23.8%)	
Christian	6 (13.6%)	9 (21.4%)	
Muslim	15 (34.1%)	21 (50%)	
Other	2 (4.5%)	2 (4.8%)	
Importance of religion			0.033*
Not important	37 (84.1%)	25 (59.5%)	
Important	5 (11.4%)	9 (21.4%)	
Very important	2 (4.5%)	8 (19.0%)	
Religiosity level			0.087
Low	29 (65.9%)	19 (45.2%)	
High	15 (34.1%)	23 (54.8%)	

*Note*: Statistical significance is indicated with * for *p* ≤ 0.05, ** for *p* ≤ 0.01 and *** for *p* ≤ 0.001.

### 3.2 Factors Affecting Religiosity Scores and Levels

Higher religiosity scores were found for HCWs (i) on COVID‐19 units, (ii) in the age group of 50–60 years old, (iii) who reported that religion was important or very important or (iv) who considered themselves Muslim. HCWs who described themselves as belonging to ‘Other’ religions had significantly lower religiosity scores than HCWs who described themselves as Jewish or Christian. In multivariable analyses, however, only experience on the COVID‐19 units or a religion classified as ‘Other’ remained significant, suggesting that these are independent risk factors for religiosity (see Table 2).

**TABLE 2:** Factors affecting religiosity total scores.

Factor	Crude		Adjusted^a^	
	*β* (95% CI)	*p*	*β* (95% CI)	*p*
Setting				
Control	(Reference)	—	(Reference)	—
COVID‐unit	1.59 (0.20, 2.97)	0.025*	1.52 (0.15, 2.89)	0.030*
Sex				
Female	(Reference)	—	(Reference)	—
Male	1.29 (−0.13, 2.71)	0.074	−0.15 (−1.70, 1.39)	0.843
Age				
20–30	(Reference)	—	(Reference)	—
30–40	−0.11 (−1.58, 1.36)	0.881	−0.08 (−1.57,1.41)	0.916
40–50	−1.96 (−4.10, 0.19)	0.074	−1.39 (−3.69, 0.91)	0.231
50–60	−5.81 (−9.55, −2.05)	0.003**	**−**3.53 (−7.24, 0.17)	0.062
Years working in COVID‐19 department				
0	(Reference)	—	(Reference)	—
< 0.5	−1.39 (−3.49, 0.71)	0.191	Not done	—
0.5–1	1.52 (−1.35, 4.39)	0.296	Not done	—
1–2	−0.19 (−2.74, 2.36)	0.882	Not done	—
2	0.62 (−1.61, 2.86)	0.583	Not done	—
Marital status				
Married	(Reference)	—	(Reference)	—
Not married	0.75 (−0.67, 2.18)	0.296	Not done	—
Children				
No	(Reference)	—	(Reference)	—
Yes	−1.06 (−2.48, 0.37)	0.144	Not done	—
Profession				
Doctor	(Reference)	—	(Reference)	—
Nurse	1.29 (−0.41, 2.99)	0.135	Not done	—
Other	1.34 (−0.46, 3.13)	0.143	Not done	—
Religion				
Judaism	(Reference)	—	(Reference)	—
Christianity	1.72 (−0.11, 3.55)	0.065	0.87 (−1.36, 3.11)	0.437
Islam	1.86 (0.43, 3.29)	0.011*	1.15 (−0.61, 2.91)	0.197
Other	−5.31 (−8.4, −2.20)	0.001***	−4.56 (−7.77, −1.34)	0.006**
Importance of religion				
Not important	(Reference)	—	(Reference)	—
Important	2.18 (0.36, 4.01)	0.019*	Not done	
Very Important	3.41 (1.31, 5.51)	0.002**	Not done	

*Note*: Statistical significance is indicated with * for *p* ≤ 0.05, ** for *p* ≤ 0.01 and *** for *p* ≤ 0.001.

^a^ Multivariable model accounted for unit, age, sex and religion to get adjusted slopes.

With marginally statistical significance, HCWs on the COVID‐19 units tended to be at greater odds for being highly religious as defined by religiosity scores > 10 (OR = 2.34; 95% CI: 0.99, 5.69; *p* = 0.056). Nurses (OR = 3.04, 95% CI: 1.04, 9.56; *p* = 0.048) and other non‐physicians (OR = 3.33, 95% CI: 1.08, 11.10; *p* = 0.041) were at significantly greater odds of being highly religious than physicians were. Muslims were also at greater odds of being highly religious (OR = 3.42, 95% CI: 1.26, 9.84; *p* = 0.020) than Jewish employees. In multivariable models, the trend for HCWs on COVID‐19 units to be at greater odds of being highly religious was no longer significant (adjOR = 2.39, 95% CI: 0.79, 7.46; *p* = 0.126) but the association still held for nurses (adjOR = 4.68, 95% CI = 1.20, 18.15; *p* = 0.029) other non‐physicians (adjOR = 9.42, 95% CI: 1.90, 46.70; *p* = 0.007). In the adjusted analysis, the association for Muslims to be at greater odds of being highly religious (adjOR = 5.87, 95% CI: 1.50, 22.99; *p* = 0.013) than Jewish employees also held. A similar, marginally statistically significant, trend was found for Christians to be more likely to be highly religious than those who described themselves as Jewish (adjOR = 5.17, 95% CI: 0.99, 26.90; *p* = 0.054) (see Table 3).

**TABLE 3:** Factors affecting religiosity levels.

Factor	Religiosity level		Odds ratios	
	Low *N* (%)	High *N* (%)	Crude	Adjusted
Setting				
Control	29 (65.9)	15 (34.1)	(Reference)	(Reference)
COVID‐unit	19 (45.2)	23 (54.8)	2.34 (0.98, 5.74)	2.39 (0.86, 6.64)^a^
Sex				
Female	29 (60.4)	21 (55.3)	(Reference)	(Reference)
Male	19 (39.6)	17 (44.7)	1.23 (0.51, 2.97)	0.73 (0.23, 2.31)^b^
Age				
20–30	19 (39.6)	17 (44.7)	(Reference)	(Reference)
30–40	18 (37.5)	18 (47.4)	1.12 (0.44, 2.83)	1.40 (0.46, 4.27)^b^
40–50	8 (16.6)	3 (7.89)	0.42 (0.08, 1.72)	0.48 (0.07, 3.26)^b^
50–60	3 (6.25)	0 (0)	Not estimable	Not estimable
Years working in COVID‐19 department				
0	7 (14.9)	7 (18.4)	(Reference)	(Reference)
< 0.5	22 (46.8)	8 (21.0)	0.40 (0.10, 1.51)	0.20 (0.04, 1.12)^b^
0.5–1	2 (4.3)	6 (15.8)	3.00 (0.49, 25.7)	1.36 (0.12, 15.45)^b^
1–2	7 (14.9)	5 (13.2)	0.71 (0.14, 3.38)	0.25 (0.02, 2.71)^b^
2	9 (19.1)	12 (31.6)	1.71 (0.42, 7.20)	0.32 (0.03, 3.93)^b^
Marital status				
Married	26 (55.3)	21 (44.7)	(Reference)	(Reference)
Not married	22 (56.4)	17 (43.6)	0.96 (0.40, 2.25)	0.95 (0.33, 2.70)^b^
Children				
No	21 (55.3)	17 (44.7)	(Reference)	(Reference)^b^
Yes	27 (57.4)	20 (42.6)	−1.06 (−2.48, 0.37)	1.76 (0.58, 5.32)^b^
Profession				
Doctor	20 (41.7)	7 (18.4)	(Reference)	(Reference)
Nurse	16 (33.3)	17 (44.7)	3.04 (1.04, 9.56)*	4.68 (1.32, 16.50)*^,^ ^c^
Other	12 (25.0)	14 (36.8)	3.33 (1.07, 11.10)*	9.42 (2.13, 41.72)**^,^ ^c^
Religion				
Judaism	22 (45.8)	9 (23.6)	(Reference)	(Reference)
Christianity	7 (14.6)	8 (21.0)	2.79 (0.77, 10.39)	5.17 (1.12, 23.95)^d^
Islam	15 (31.3)	21 (55.3)	3.42 (1.26, 9.84)*	5.87 (1.65, 20.88)*^,^ ^d^
Other	4 (8.3)	0	Not estimable	Not estimable

*Note*: Statistical significance is indicated with * for *p* ≤ 0.05 and ** for *p* ≤ 0.01.

^a^ Adjusted for profession and religion.

^b^ Adjusted for unit, profession and religion.

^c^ Adjusted for unit and religion.

^d^ Adjusted for Unit and Profession.

### 3.3 Impact of Unit and Religiosity on Self‐Measures of Performance

No significant effect of religiosity level, unit or interaction of religiosity level with unit was detected on GAD‐7, PSQI, CD‐PRS and CCRAM scores. Further examination of these dependent variables revealed that personal resilience (CD‐PRS) scores were significantly affected by age, with younger, 30 to 40‐year‐old employees (30–40; *p* = 0.016) and older (50–60) adults having more resilience (*p* = 0.023). HRQ scores were significantly lower for males (*β* = −5.79, 95% CI = −9.57, −2.02; *p* = 0.003) and tended to be higher for individuals with children (*β* = 3.45, 95% CI: −0.47, 7.37; *p* = 0.084). Moreover, HRQ scores tended to be lower for Christians (*β* = −4.91; 95% CI: −10.46, 0.64; *p* = 0.082) and significantly lower for Muslims (*b* = −5.81; 95% CI: −10.13, −1.49; *p* = 0.009). PSQI tended to be lower for Christians (*β* = −4.37, 95% CI: −8.8, 0.071; *p* = 0.053) and higher for nurses (*β* = 3.22, 95% CI: −0.44, 6.90; *p* = 0.084) but results did not reach statistical significance.

**FIGURE 2:**
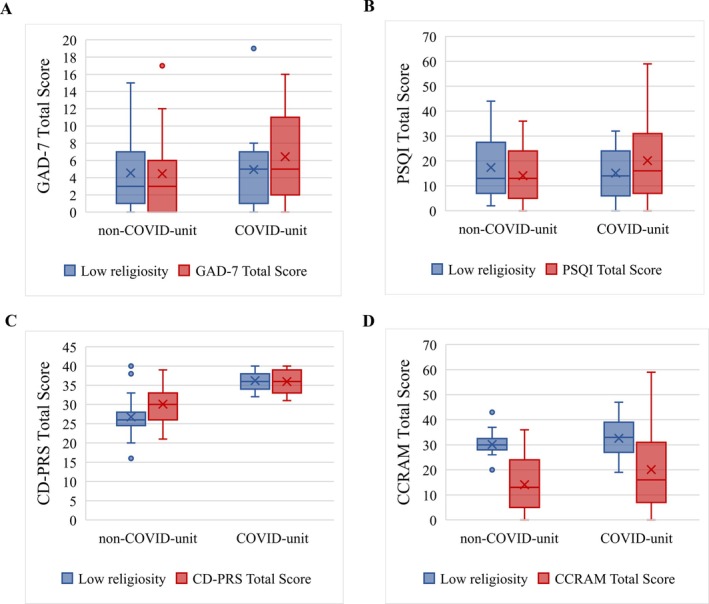
Impact of assigned unit (non‐COVID vs. COVID) and religiosity level on self‐reported measures of anxiety (A), sleep quality (B), personal resilience (C) and community resilience (D).

To adjust for potential confounders, a series of multivariable analyses was performed. When personal resilience (CD‐PRS) scores were adjusted for age, the interaction between unit (non‐COVID vs. COVID) and religiosity level was marginally statistically significant (*p* = 0.061). Individuals with high religiosity tended to be more resilient on the control units but less resilient on the COVID‐19 units. Adjustment for other potential confounders had no effect on the results for HRQ or PSQI, and no potential confounders were found for GAD‐7 scores (see Figure 2).

### 3.4 Impact of Religiosity Level on ASAT Outcomes for COVID‐19 and Non‐COVID‐19 Health Care Workers

**FIGURE 3:**
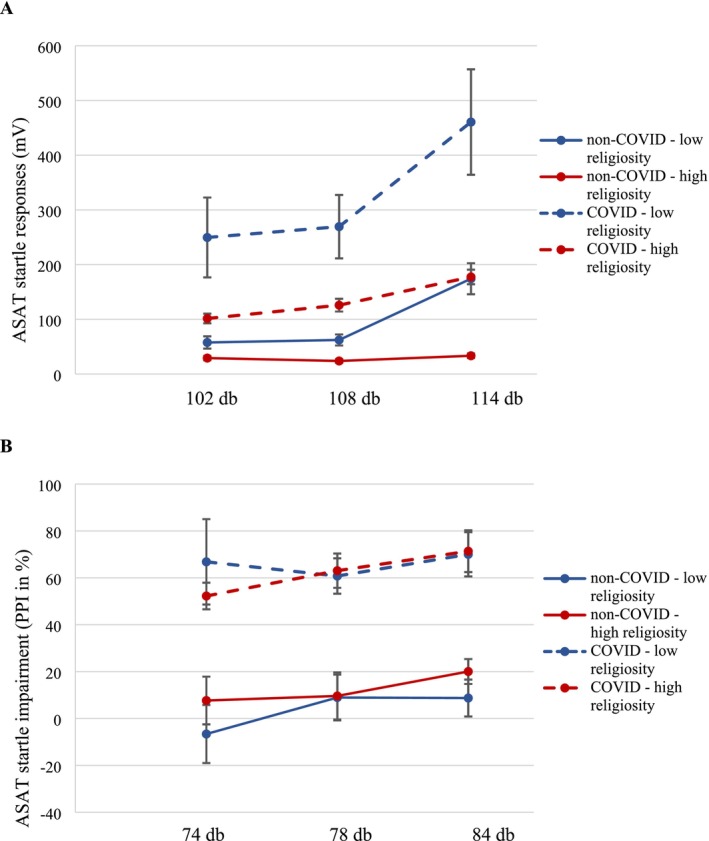
Impact of assigned unit (non‐COVID vs. COVID) and religiosity level on startle responses (A) and pre‐pulse inhibition of 114 db acoustic startles (B) measured with the Auditory Sustained Attention Test (ASAT).

Auditory stimuli at higher decibel levels led to increased ASAT startle responses (*F*_2,136_ = 53.39; *p* < 0.001). A significant interaction between unit and religiosity level (*F*_1,68_ = 4.21; *p* = 0.044) was detected. In general, the HCWs in the high religiosity group tended to respond with a lower amplitude of the ASAT startle response (see Figure 3A). Further analysis revealed that HCWs with low religiosity on the COVID‐19 units had a 2‐fold higher (*β* = 2.02, 95% CI: 1.07, 3.84) acoustic startle responses than HCWs with low religiosity on non‐COVID‐19 units (*p* = 0.030). No significant differences were detected between COVID and non‐COVID HCWs in the high religiosity group.

The interaction for religiosity level and decibel level of the stimulus approached statistical significance (*F*_2,136_ = 2.61; *p* = 0.077). This was due to HCWs in the low religiosity groups having a 1.65‐fold (95% CI: 1.14, 2.41; *p* = 0.008) greater difference between the 114 and 102 db stimuli than those with high religiosity. No impact of sex, profession, marital status, children or religion was detected, nor did these factors affect the dose response to the acoustic startle.

Moreover, the HCWs working on the COVID‐19 units showed distinctive patterns of attention dysregulation, manifesting as a hypervigilance pattern on the ASAT, along three prepulse auditory stimuli (namely 74, 78 and 84 dB) leading to 60%–70% PPI inhibition rates (*F*_1,72_ = 44.78, *p* < 0.00001, *η* ^2^ = 0.383) (see Figure 3B).

## 4 Discussion

This study investigated self‐reported and neurophysiological mechanisms of resilience among HCWs exposed to the high‐stress environments of COVID‐19 units, versus those working on non‐COVID units. We found that working on COVID‐19 units was associated with significantly elevated emotional dysregulation—based on exaggerated startle responses—and attention dysregulation with hypervigilance patterns—based on increased PPI > 50%—as measured with the ASAT. As the ASAT measures the eyeblink reflex to sudden startle and pre‐pulse auditory stimuli, it is considered an objective measurement of both emotional and (sustained) attentional dysregulation mechanisms (Cohen et al. 2024). With increased decibel levels, an increasing response is expected, and this was observed in the current study. For HCWs working under the heightened stress of COVID‐19 units, however, there was an exaggerated response compared to HCWs exposed to less stressful environments. Of note, HCWs did not report any significantly heightened levels of anxiety on the GAD‐7.

A previous study found—similar to the current study—that physicians working on COVID‐19 units experienced a significant elevation of physiological measures of anxiety and attention vigilance (measured with the ASAT) 3 months into the pandemic (Dolev et al. 2021). The current study adds that these patterns of physiological hypervigilance and anxiety are found in both physicians and in other HCWs in the COVID‐19 units, including nurses and administrative staff. Furthermore, we show that physiological measures can distinguish anxiety and vigilance levels between HCWs in COVID‐19 versus non‐COVID‐19 units, even when self‐reported measures do not (yet) significantly differ. One conclusion would be that the ASAT can detect symptoms that are risk factors for later onset of MHP, such as PTSD, before they are detectable with measures from traditional, self‐reported instruments. This is important to note as it elucidates potential pathways to objectively screen at‐risk populations for early MHP by using objective neurophysiological tools such as the ASAT.

### 4.1 Religion and Resilience

Our study aimed to demonstrate the difference in resilience between HCWs who report high levels of religiosity versus those who report low levels of religiosity. It was found that working on a COVID‐19 unit or a religion classified as ‘Other’ were independent risk factors for high religiosity. HCWs who reported low religiosity scores showed heightened emotional and attentional dysregulation as measured with the ASAT, and reported lower levels of personal resilience on the CD‐PRS. However, no differences on the self‐reported anxiety (GAD‐7) or sleep quality (PSQI) were reported. These findings thus confirm the proposed hypothesis of the study, being that high levels of religiosity are associated with higher levels of personal resilience and better mechanisms of emotional regulation (i.e., showing better neurophysiological resilience mechanisms). When interpreting these results, it is important to acknowledge the complexity of measuring resilience as it is a multidimensional construct influenced by various psychological, social, cultural and environmental factors (Rutter 2012; Windle 2011). Resilience encompasses a dynamic interplay of individual traits (e.g., self‐efficacy and emotional regulation), social support systems, community cohesion and broader cultural and environmental contexts (Fletcher and Sarkar 2013; Rutter 2012; Windle 2011). Similarly, religiosity is a multifaceted construct that includes cognitive (religious beliefs), behavioural (religious practices) and emotional (sense of connection) dimensions (Pargament 1997). The HRQ used to assess religiosity in this study consists of three questions that broadly encompass these dimensions. While each question covers one of the religiosity dimensions, it may not fully encompass the nuances of religious belief, practice or community engagement.

The results of our study do align with the limited number of studies investigating the effect of religion on psychological resilience in HCWs during the COVID‐19 pandemic. As mentioned in the introduction section of this paper, it has been previously shown that religious practice may enhance resilience and stress‐adjustment (Ano and Vasconcelles 2005) and that in the context of the COVID‐19 pandemic religion is associated with reduced levels of mental distress (Chang et al. 2021; Mesri et al. 2022), reduced anxiety levels (Tolentino et al. 2022) and increased psychological resilience (Angin 2021). Like previous studies, this study found a high level of religiosity to be associated with enhanced resilience and reduced—neurophysiological, not self‐reported—anxiety.

Finally, it is interesting to note that although the ‘high’ and ‘low’ religiosity groups showed similar total community resiliency scores, the ‘low’ religiosity group showed higher scores on the specific item in the CD‐PRS ‘My settlement is well‐organized for emergencies, including the COVID‐19 crises’. Although the difference on this specific item could be influenced by many different factors, one could hypothesis that it is the very nature of a high level of religiosity, specifically the Jewish and Muslim faith, which may cause the community to feel less equipped for emergencies such as a pandemic. Both Judaism and Islam are characterised by high levels of community involvement and their religious requirements include set prayers (3‐ and 5‐times daily respectively), preferably in synagogues or mosques. Both religions put a strong emphasis on communal prayer and activities that were heavily and adversely impacted during the COVID‐19 pandemic. Although adaptations were made by religious leaders to incorporate religious practice within the ‘new reality’ of the pandemic (Frei‐Landau 2020), the sense of belonging and experience of community resilience were nonetheless compromised.

## 5 Limitations and Future Directions

One limitation of this study is its cross‐sectional design and the lack of data about the mental health of the HCWs prior to the study. Thus, causal statements and the longer trajectory of religiosity, resilience and MHP cannot be derived from the study. Insights into the causal and long‐term effects would require the conductance of longitudinal studies. These, however, would run into other problems in terms of preserving subject anonymity and the Hawthorne effects, where people participating in a study about religiosity may start attending services and praying more.

Furthermore, religious coping—a specific concept that focuses on how individuals use their religious beliefs and practices to cope with stressful life events—was not specifically addressed in this research. Previous research established that religious coping can be categorized into positive and negative forms, each associated with either positive or negative psychological adjustment (Ano and Vasconcelles 2005). In the current study, this differentiation was not investigated, which could potentially have influenced the data. It should also be noted that the HRQ only consists of three questions to establish the religiosity levels, so some care should be taken regarding the exact meaning and implications regarding the religiosity, for example, communal activity and daily life function. Future studies should further differentiate between the exact meaning of the religiosity, the involvement in religious‐group‐related activities and the general values tied to the religious beliefs (whether positive or negative, supportive or punitive, etc.). Additionally, future research should further explore religious coping as a separate construct in addition to religiosity and resilience to better understand its potential role in psychological adjustment.

Finally, due to the predominance of Muslim and Jewish HCWs (respectively 41.9% and 36.0%) as well as the specific circumstantial characteristics of country, culture, workload and pandemic‐related measures, the generalizability of the results may be limited. Future studies should examine the correlation between religion and resilience in a broader or different setting, for example with HCWs with different religious traditions or within different hospital settings (academic, general, peripheral, etc.). This would further the general understanding of how the level of religiousness may contribute to resilience against MHP among HCWs.

## 6 Conclusions

In summary, this study found that HCWs exposed to the high‐stress environments of COVID‐19 units showed significantly elevated levels of emotional and attentional dysregulation but did not report a significantly heightened level of anxiety on the GAD‐7 as compared to HCWs at non‐COVID units. Findings confirmed that HCWs with a high level of religiosity found protection for their emotional regulation and personal resilience through their level of religious values. These findings may lead to novel pathways to objectively screen at‐risk populations for early MHP, including posttraumatic symptoms and anxiety, and indicate religiosity as a target to increase resilience.

## Ethics Statement

All procedures were in accordance with the ethical standards of the institutional research committee at [anonymized for review] and with the 1964 Helsinki Declaration and its later amendments or comparable ethical standards.

## Consent

Informed consent was obtained from all individual participants included in the study. [Anonymized for review] is Mindtension (ltd) consultant.

## Conflicts of Interest

Avi Avital is Mindtension (ltd) consultant.
